# The Application of Biometric Approaches in Agri-Food Marketing: A Systematic Literature Review

**DOI:** 10.3390/foods12162982

**Published:** 2023-08-08

**Authors:** Lei Cong, Siqiao Luan, Erin Young, Miranda Mirosa, Phil Bremer, Damir D. Torrico

**Affiliations:** 1 Department of Agribusiness and Markets, Lincoln University, Lincoln 7647, New Zealand; 2 New Zealand Food Safety and Science Research Centre, Palmerston North 4474, New Zealand; s.luan@qub.ac.uk (S.L.); erin.young@otago.ac.nz (E.Y.); miranda.mirosa@otago.ac.nz (M.M.); phil.bremer@otago.ac.nz (P.B.); 3 Department of Marketing, University of Otago, Dunedin 9010, New Zealand; 4 Queen’s Management School, Queen’s University Belfast, Belfast BT9 5AH, UK; 5 Department of Food Science, University of Otago, Dunedin 9010, New Zealand; 6 Department of Wine, Food and Molecular Biosciences, Lincoln University, Lincoln 7647, New Zealand; damir.torrico@lincoln.ac.nz

**Keywords:** biometric, agri-food, eye-tracking, marketing, consumer behaviour

## Abstract

A challenge in social marketing studies is the cognitive biases in consumers’ conscious and self-reported responses. To help address this concern, biometric techniques have been developed to obtain data from consumers’ implicit and non-verbal responses. A systematic literature review was conducted to explore biometric applications’ role in agri-food marketing to provide an integrated overview of this topic. A total of 55 original research articles and four review articles were identified, classified, and reviewed. It was found that there is a steady growth in the number of studies applying biometric approaches, with eye-tracking being the dominant method used to investigate consumers’ perceptions in the last decade. Most of the studies reviewed were conducted in Europe or the USA. Other biometric techniques used included facial expressions, heart rate, body temperature, and skin conductance. A wide range of scenarios concerning consumers’ purchase and consumption behaviour for agri-food products have been investigated using biometric-based techniques, indicating their broad applicability. Our findings suggest that biometric techniques are expanding for researchers in agri-food marketing, benefiting both academia and industry.

## 1. Introduction

Food marketers are always keen to improve the methods they use to study consumers’ behaviours and achieve marketing objectives by better understanding consumers’ perceptions. Traditionally, direct measurements, such as self-reports, surveys, focus groups, or interviews, have been widely used [1]. However, one significant weakness of direct measurements is that they rely on conscious and self-reported responses, which risk being affected by cognitively determined factors [2]. Since traditional consumer insights data may have cognitive biases, they may not be robust enough to help industries fine-tune their food packaging or advertising messages to ensure they resonate well with the targeted consumers.

Recently, to decrease cognitive biases and enhance the understanding of consumers’ sub-conscious behaviours when faced with apparently “real-life” food-related decision-making, biometrics have been gradually recognised and employed by marketing researchers [3] as an essential tool to assess consumers’ responses by measuring either physiological characteristics such as heart rate, body temperature, or skin conductance or behavioural ones such as subconscious emotions and eye movements [4]. Compared with data collected from self-reported responses, surveys, interviews, etc., data from biometric approaches are based on the analysis of consumers’ implicit and non-verbal responses and, consequently, can reflect consumers’ sub-consciousness, e.g., preferences, attitude, etc., without cognitive influences [3].

### 1.1. Defining Biometrics

The term biometrics derives from the Greek word “bios” (life) and “metro” [5]. As stated by Corcoran, Sims, and Hillhouse (1999) [6], biometrics is used to measure individuals’ unique characteristics and use those clues to characterise them. For this study, biometrics refers to “the methods that may be used in humans or animals to identify or recognise their physiological and behavioural distinctive characteristics” [7].

As per previous studies, any physiological or behavioural attribute, e.g., face-related movements and skin-related changes, can qualify as being a biometric trait unless it satisfies the criteria such as: (i) universality: possessed by all humans; (ii) distinctiveness: discriminative amongst the population; (iii) invariance: the selected biometric attribute must exhibit invariance against time; (iv) collectability: easily collectible in terms of acquisition, digitisation, and feature extraction from the population; (v) performance: pertains to the availability of resources and imposition of real constraints in terms of data collection and a guarantee to achieve high accuracy; (vi) acceptability: the willingness of the population to submit that attribute to a recognition system; (vii) circumvention: prone to imitation or mimicry in case of fraudulent attacks against the recognition system [8,9].

### 1.2. Biometrics Usage

Consumers are already familiar with biometric approaches as an important identification tool in their daily lives. For instance, they often use face and fingerprint biometrics to access their mobile devices and unlock doors [10]. Additionally, consumers encounter biometric technologies in various industries that simplify their experiences. For example, facial recognition kiosks allow self-check-in at hotels [11] or check-out after shopping [12], and biometric identification facilitates easy access to medical information in healthcare centres [13]. 

Moreover, some businesses are gaining traction to use biometrics to promote their products and services. A notable example is a car manufacturer that uses facial expressions and heartbeat to predict a driver’s emotions, thereby personalising the driving experience with customised lighting, sound, cabin temperature, seat vibration, and scent [14].

However, it is essential to note that biometrics can be used as a research tool to gain consumer insights on a deep level [10,15,16]. For a marketer, the benefit of employing biometrics is enormous. Sheth and Kellstadt (2021) [17] even call biometrics data one of the next frontiers of research in marketing. As previously mentioned, the main benefit of employing biometrics technologies is that they could deliver a level of consumer understanding previously unachievable with traditional research methods [10]. Thus, biometrics technologies based on a wide range of physiological or behavioural attributes, e.g., eye movements, skin conductance, heart rate, emotion changes, and facial changes, have been used for marketing [18].

Although biometrics has already become pervasive in many applications [19], its adoption by marketing researchers is progressing relatively slowly, including in agri-food marketing. Hence, a better understanding of the past, current, and potential utilisation of biometric approaches in agri-food marketing will be beneficial, and it may help to introduce such techniques and improve research and marketing practices. Therefore, this study systematically reviewed biometric approaches’ utilisation in agri-food marketing. Stemming from this aim, the authors explored and provide insights by addressing the following research questions:

RQ 1. What is the current status of utilising biometric approaches in marketing studies in the agri-food sector?
RQ 2. How have biometric approaches been used or studied in agri-food marketing?

## 2. Methodology and Data Acquisition

To address our research questions, this review article is organised as follows: Section 2 introduces the systematic literature review methodology and how it was adopted in this study to identify extant research in biometrics application in agri-food marketing. Section 3 illustrates our findings by answering the research questions. We specifically reviewed the current literature on utilising biometric approaches in the agri-food marketing sector and summarised how biometric approaches have been used. Section 4 discusses the advantages and disadvantages of utilising biometric techniques and presents the direction for future application avenues. Finally, a conclusion is reported in Section 5.

To generate an up-to-date overview of existing research on biometric approaches in agri-food sector marketing, we conducted a systematic literature review, considered the most-appropriate tool for systematically assessing and evaluating a given body of literature [20]. In addition, a systematic literature review is an effective method to identify existing gaps, which could provide instructions for future research. Data for our systematic literature review were extracted and gathered from Google Scholar (https://scholar.google.com/) (accessed on 1 September 2022), one of the most-comprehensive sources of indexed academic publications. Google Scholar was chosen over other sources because of its broad coverage and robust search algorithm. Gusenbaur (2019) [21] commented that Google Scholar provides the most-exhaustive range of available resources after comparing Google Scholar with 11 other search engines and bibliographic databases.

### 2.1. Article Selection

The data collection process was designed based on methods established by previous articles [22,23]. Firstly, to search the database, a set of keywords related to biometric approaches was first identified. A query using a combination of these keywords (adopting the Boolean operators “OR” and “AND”) was run in the fields related to “title”, “abstract”, and “keywords”. Works published up until 30 September 2022 were included. The keywords were combined in the following string to search: (biometric* OR eye-tracking OR skin-conductance OR “heart rate” OR facial OR emotion-recognition OR “facial decoding” OR “implicit measurement” OR electroencephalography OR emotion OR “physiological measures” OR “facial electromyography” OR “facial expression” OR “EEG” OR “ECG” OR electrodermal OR attention OR “galvanic skin response” OR “GSR” OR “automated facial expression analysis” OR “AFEA”) AND (marketing OR consumer OR perception OR acceptance OR behavior? OR food OR agri-food). 

For the first step of the selection process, identification, a sensitivity rather than specificity approach was adopted, meaning including articles rather than excluding them [24]. The initial literature search resulted in 8735 records. The authors included all the papers for the next selection step: eligibility screening. Eligibility screening of the records was performed based on inclusive and exclusion criteria (see Table 1). Edwards et al. (2002) [25] proposed that an average of 8% of eligible studies would be missed if one person did the eligibility screening. In contrast, no studies would be missed if two people conducted the eligibility screening. Therefore, the eligibility screening was conducted by two authors to ensure that all qualified articles focusing on applying biometric approaches in the agri-food sector have been included. Disagreement was resolved by discussion between the first two authors to ensure all selected papers met the inclusion criteria in Table 1. 

**Figure 1:**
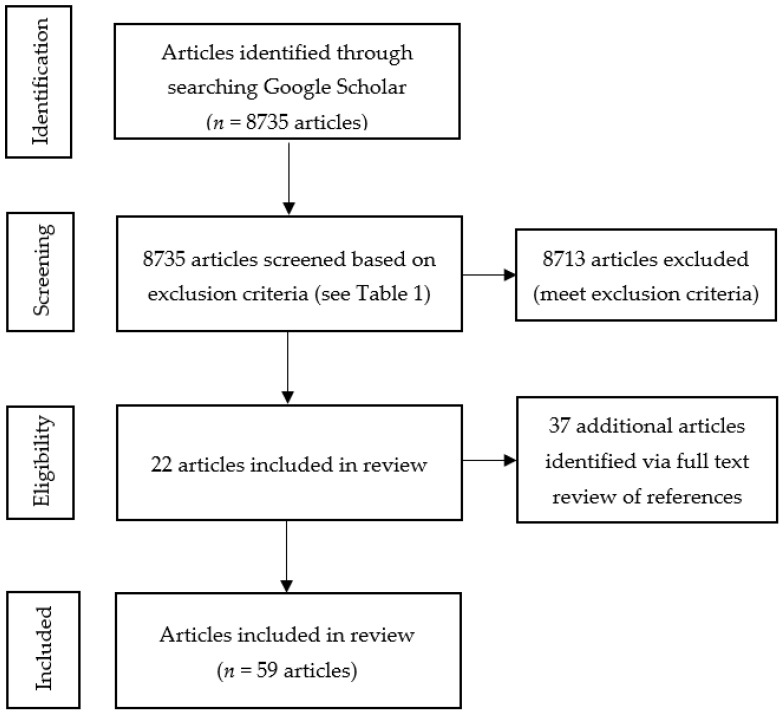
Flow chart of different phases of the systematic review.

In the end, after excluding proceedings, opinion papers and outlooks, conference papers, abstracts, concept articles, and materials not published in English and confining the search to the application of biometric approaches in the agri-food marketing sector, 22 articles were included in the systematic review. By checking the reference lists of the studies cited, an additional 37 papers were identified that met the inclusion criteria. In total, 59 articles, including four review articles [7,26,27,28] and 55 original research articles, were included in the final review. A flow chart summarising the study selection process is depicted in Figure 1.

### 2.2. Data Collection

**Figure 2:**
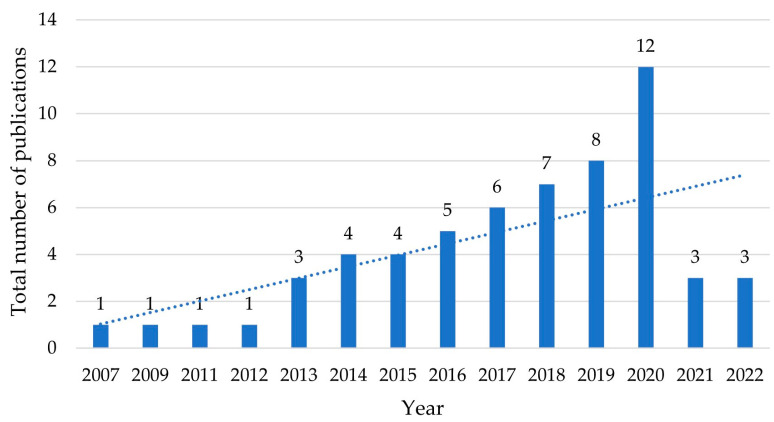
Number of selected articles by year of publication (*n* = 59).

**Figure 3:**
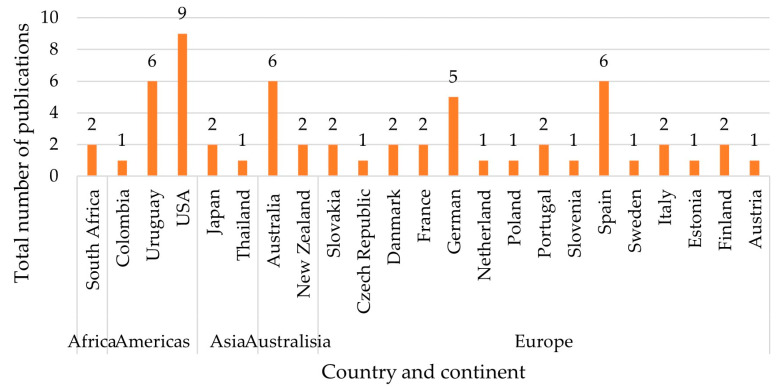
Geographical distribution of selected articles (*n* = 59).

**Figure 4:**
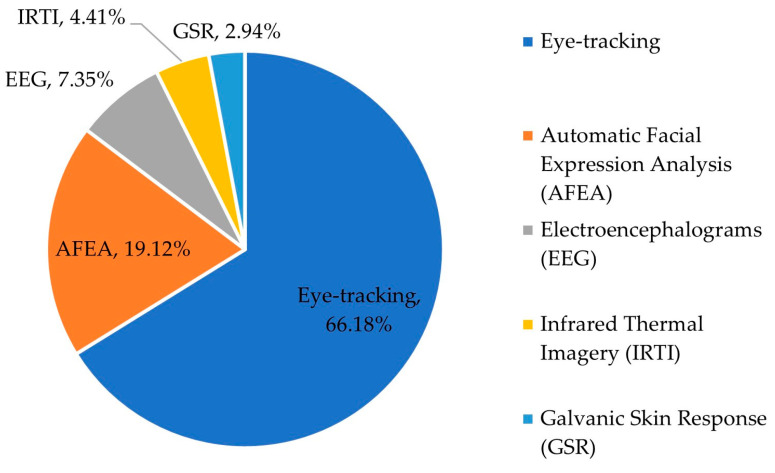
Distribution of utilisation frequency in selected research articles (*n* = 55).

In the next step, the second author extracted the data from the included articles, and the corresponding author checked the extracted data. Information was collected from each study on (1) the year of publication, (2) the geographic location of study samples, (3) the utilised biometric approaches, (4) the data collection types, (5) the food products, and (6) the primary outcomes regarding food marketing. The summary information of the articles is shown in Figure 2, Figure 3 and Figure 4 and Table 2 and Table 3.

## 3. Analysis and Findings

As an initial step toward understanding the nature of the current literature, we first provide the key descriptive statistics of the selected article corpus to answer RQ1 (What is the current status of utilising biometric approaches in agri-food sector marketing?). Second, we examine how biometric approaches have been used in agri-food marketing (RQ2) by categorising different scenarios, e.g., consumers’ general perceptions of a food product, reading food packaging, or shopping in retail shops.

### 3.1. Current Status of Utilising Biometric Approaches in Agri-Food Sector Marketing

#### 3.1.1. Chronological and Demographical Analyses

Chronologically, the trend in the number of included research articles was positive (Figure 2), demonstrating a growing interest in and use of biometrics applications among researchers in the field of agri-food marketing. Biometric technologies have only been fully appreciated by researchers in the last decade. Specifically, the first included research article was published in 2007. After that, an increase in the number of published articles was not seen for the following four years, indicating a starting stage of studies in this area. From 2013 to 2020, there was a steady upward trend in articles published, from 1 in 2012 to 12 in 2020. 

However, in the two final years under investigation, i.e., from 2021 to 2022, the number of articles decreased dramatically. A possible reason behind the sharp decline was the adverse impact of the COVID-19 pandemic on scientific research. Several studies have reported that the COVID-19 pandemic has undoubtedly disrupted the scientific enterprise, for example the decline in research time, especially for scientists working in physical laboratories [29] and the disruption of the reviewing and publishing process for research articles [30]. Considering the facility requirement of biometrics studies and the timeline of publishing research outcomes, the relatively small number of studies we can find in 2021 and 2022 is not surprising.

A geographical analysis of the selected articles indicates how widespread biometrics applications are in the agri-food sector (see Figure 3), with a total of 23 countries represented. Note that the geographic location was analysed based on where the data were collected. Multiple countries were counted if the study was conducted across different countries. The affiliations of the first author were used when data collection locations were not introduced in some articles. According to the analysis, the largest share, 30 studies, was conducted in Europe. Of these, the highest number of studies was conducted in Spain (6 articles) and Germany (5 articles). At the same time, Slovakia, Denmark, Portugal, France, Italy, and Finland presented two studies, respectively, and Austria, the Czech Republic, Netherlands, Poland, Slovenia, Sweden, and Estonia contributed one each. Undoubtedly, the most-research-active country in this area is the United States of America (USA), with nine publications included in the current review. Other continental American countries included are Uruguay (6 articles) and Colombia (1 article). Two Australasian countries, Australia and New Zealand, have also played a role in this area, with six and two publications, respectively. Although Japan (2 articles), South Africa (2 articles), and Thailand (1 article) have contributed some research on this topic, the research appears to be limited in Asian and African countries in general.

#### 3.1.2. Approaches Utilised

According to the selected papers, the biometric technologies that marketing researchers within the agri-food sector have adopted include eye-tracking, automatic facial expression analysis (AFEA), electroencephalogram (EEG), galvanic skin response (GSR), and infrared thermal imagery (IRTI). The pie chart below (see Figure 4) displays the distribution of how many times they have been utilised in the selected articles. Table 2 summarises the rationale of how these methods can be used as marketing tools to understand consumers. 

Specifically, eye-tracking has been more commonly used across the included research articles in the corpus (66.18%). Eye-tracking is a non-invasive and promising technique to explore gaze patterns of visual attention [31]. Eye-tracking can measure eye movements (fixation and saccades) and pupil dilation, determining precisely where the users’ attention is directed. Awareness can indicate purchase intent [32], so monitoring eye movements could effectively explore factors influencing consumers’ buying intention. Eye-tracking is widely used in consumer behaviour studies, assessing consumers’ perceptions of visual stimuli, such as food packaging and labels [33,34]. Detailed information about how eye-tracking is used will be presented in Section 3.2.

Among the biometric approaches, the second-most-common technique is automatic facial expression analysis (AFEA) (19.12%). AFEA is a technique for measuring emotional valence based on the movements of facial features, which relies on the assumption of coherence between emotion and facial expressions [35]. It focuses on translating combinations of facial movements (e.g., lips, eyes, cheeks, and mouth movements) to classify and provide an estimated intensity for the six basic emotions defined by Ekman (1993) [36], happiness, sadness, surprise, fear, anger, and disgust.

The next most-used biometric approach is electroencephalograms (EEGs), accounting for 7.35%. EEG is a non-invasive instrument widely used to measure consumers’ brainwaves before, during, and after exposure to stimuli. It measures consumers’ cortical activation by detecting electrical activity through EEG electrodes on the scalp’s surface [37]. In this sense, EEG is superior in collecting real-time data, which could assist researchers in better comprehending causal connections between the marketing stimuli and corresponding cognitive responses [38]. 

Studies using the techniques of either galvanic skin response (GSR) or infrared thermal imagery (IRTI) were only used within a few agri-food marketing articles (2.94% and 4.41%, separately). GSR, which is also known as electrodermal activity (EDA), is a way of measuring people’s skin conductance level (SCL), short-duration skin conductance responses (SCRs), and heart rate (HR) [39]. The SCL, SCRs, and HR are all important indices that reflect consumers’ internal emotional states when viewing food products. These data allow marketers to overcome cognitive biases and comprehend consumers’ thoughts. IRTI is a device capable of capturing “the heat emitted by the subject of the surveillance and forms an image using IR radiation” [40]. With the help of IRTI, researchers can figure out participants’ natural reactions towards food stimuli based on changes in body temperature.

In this sense, all the abovementioned biometric approaches can help marketers have a better understanding of consumers’ underlying cognitive thinking.

### 3.2. How Have Biometric Approaches Been Used in Agri-Food Marketing?

Biometric approaches are widely used to capture consumers’ implicit perceptions during different stages of shopping or consuming food products. The different research contexts in which biometrics have been applied were categorised into five groups. The first, Section 3.2.1, was to capture consumers’ responses while watching promoted information. The second (Section 3.2.2) caught consumers’ reactions when wandering in retail shops. The third (Section 3.2.3) scenario captured consumer reactions to food packaging. The fourth (Section 3.2.4) captured consumers’ responses while reading food labels. Finally, consumer reactions to consuming food products were caught in the fifth (Section 3.2.5) scenario. Table 3 summarises the critical content from this section’s 55 collected original research articles. 

**Table 3:** Articles illustrating consumers’ responses in different scenarios of shopping or consuming food products.

Author/Year	Food Product	Biometric Approach	Main Outcome
**Scenario: watch promotion information**			
Stockburger et al. (2009) [41]	Meat dishes, vegetable dishes, and desserts	EEG	Meat stimuli are efficient attention catchers in vegetarians.
Hummel et al. (2017) [42]	Cake, orange, corn, and hamburger	Eye-tracking	Cut-up, ready-to-eat, low-calorie food attracted more visual attention than unprepared, low-calorie, and high-calorie food. Men were found to pay more attention to high-calorie food, while women paid more attention to low-calorie food.
Viejo et al. (2018) [43]	Beer	AFEA, eye-tracking, and IRTI	Consumers prefer beers with medium foam height and consider beers with low foam as non-desirable.
Motoki et al. (2018) [44]	Snacks, fruits, candies, salads, noodles, burgers, etc.	Eye-tracking	The hedonic components of foods could capture automatic visual attention more effectively.
**Scenario: wander in retail shops**			
Reutskaja et al. (2011) [45]	Junk food such as candy and chips	Eye-tracking	Consumers are good at optimising within a set of items they see during the search process. Consumers’ search process is random concerning the value, and products with a higher value are not more likely to be noticed.
Mitterer-Daltoé et al. (2014) [46]	Fish products such as fillets, nuggets, and fish burgers	Eye-tracking	Consumers focused more on a “new or different presentation” to decide whether the dish was more or less healthy; however, unusual presentations and fried products were perceived as less healthy.
Banović et al. (2016) [47]	Red meat products	Eye-tracking	Visible fat had a negative relationship with consumer perception.
Jaeger et al. (2018) [48]	Apple	Eye-tracking	Food products’ appearance was among the most-important factors influencing consumers’ purchase intention.
Clement (2007) [49]	Pasta and jam	Eye-tracking	Consumers exhibited a muddled search strategy when shopping in grocery shops. Packaging design influences consumers’ in-store buying decision process in several phases.
Vriens et al. (2020) [50]	Snacks such as nuts, cookies, chips, cereal bars, and beef jerky	Eye-tracking	Directional cues, such as promotion, significantly affect consumers’ attention and increase the chance that a consumer will decide to buy.
Zuschke (2020) [51]	Chocolate bars	Eye-tracking	Products’ positioning on a display shelf could draw consumers’ attention. Furthermore, the size and visual salience of products on shelves could attract consumers’ attention and influence their choices.
Monteiro et al. (2020) [52]	Wine	Eye-tracking	Consumers’ attention paid to the wine bottle is a determinant of their purchase intention. Furthermore, quality perceptions and desire significantly influence consumers’ wine-purchase intentions.
Bialkova et al. (2020) [53]	Muesli bars	Eye-tracking	The brand’s strength and product variety determine where consumers’ attention goes and, thus, the purchase decision. Products’ placement emerged as a significant determinant in the in-store environment. Nutrition labels increased attention and influenced purchase outcomes, but these effects were contingent on the purchase goals. Colour-coded labels and placement further lifted attention and purchases, but the brand’s strength modulated these effects. Shopping goals and prior knowledge also played a role in determining which product gets the most attention and is often chosen.
Gidlöf et al. (2017) [54]	Pasta, cereal, and yogurt	Eye-tracking	Product packages and displays could catch consumers’ attention; display attributes included the number of facings, visual salience, and product placement.
Drexler and Souček (2017) [55]	Packed vegetables, dairy food, packaged fish, packaged meat, and frozen food	Eye-tracking	Shelf level significantly influences the variability of attention of all product categories, except vegetables. The influence of the type of shelves was proven for meat and fish.
**Scenario: look at food packaging**			
López-Mas et al. (2022) [56]	Fish products	Eye-tracking, AFEA, and GSR	Involving consumers in all stages of the NPD is recommended, especially in packaging design, as it has been proven that co-creation is a straightforward and effective way to design the fish product’s packaging according to consumers’ needs and demands.
Horská et al. (2020) [57]	Cheese	Eye-tracking	Consumers pay attention to factors such as brand name, type of product, specific features, the origin of ingredients, and the aesthetic side of design when they look at product packaging.
Jantathai et al. (2013) [58]	Cake	Eye-tracking	The colours of the used food products significantly affected the gazing behaviour and the choice.
Varela et al. (2014) [59]	Breakfast cereal	Eye-tracking	When there was abundant information on food packages, colour was one of the factors attracting most of the consumers’ attention, together with brands, product names, and graphics. In contrast, consumers noticed less health information.
Husić-Mehmedović et al. (2017) [60]	Beer	Eye-tracking	Physical and semantic package features affect attention during the “orientation” phase and reveal how efficiently attention is transferred to the brand in the “discovery” phase. Moreover, packages that attract the most attention are not necessarily likeable or suitable, but recall is also a questionable measure of attention.
Clark et al. (2021) [61]	Milk	AFEA	Yellowish colour, decorative fonts, and curved shapes are well associated with happiness feeling and purchasing intention.
García-Madariaga et al. (2019) [62]	Soft drinks and snacks	Eye-tracking and EEG	The presence of visual elements, either images or texts on packages, increased the participants’ level of attention. Colour modifications do not significantly affect participants’ neurophysiological attention levels. Furthermore, the neurophysiological effects among the participants do not necessarily coincide with their subjective evaluations of preference.
Zhang and Seo (2015) [63]	Donuts, tacos, and cake	Eye-tracking	Background salience did affect participants’ visual attention. Specifically, the more complex and salient the table setting and decoration, the less attention consumers pay to the food items. The image location is an essential factor to be studied as well.
Rebollar et al. (2015) [64]	Chocolate snack	Eye-tracking	Consumers prefer the logos in the upper left corner of the packaging.
Vergura and Luceri (2018) [65]	Lemon cake, biscuits, and focaccia snack	AFEA	Compared to the background representation, the foreground representation of products elicits higher favourable emotional reactions because there is less perceived psychological distance between the subject and the product. However, the purchase intention did not significantly differ between the foreground and background conditions.
Lacoste-Badie et al. (2020) [66]	Biscuit	Eye-tracking	Front-of-pack variations catch respondents’ attention. Visual salience, such as colour, shape, and motion, can capture consumers’ attention.
Fazio et al. (2020) [67]	Olive oil	Eye-tracking	Olive oil packages with a hand using the product could attract consumers’ attention.
Hurley et al. (2015) [68]	Fruit drinks	Eye-tracking	There were no significant differences in consumers’ attention when they shopped for fruit drinks with digital or flexographic labels.
Samant et al. (2018) [69]	Potato chips product	Eye-tracking	Food neophobia will affect consumers’ expected and actual likings of ethnic-flavoured potato chips, especially Chinese-flavoured ones.
Gunaratne et al. (2019) [70]	Chocolate	Eye-tracking, AFEA	Fixations on familiar chocolate packaging were correlated with happiness. The study intended to ascertain how culturally distinct consumers from Northern Europe and Northeast Asia perceive and choose product packaging differently.
Ploom et al. (2020) [71]	Biscuit	Eye-tracking and AFEA	European and Asian consumers have different perceptions of packaging design and choices, and the authors suggested marketers adapt packaging design accordingly.
**Scenario: read food labels**			
Ares et al. (2014) [72]	Yogurt	Eye-tracking	Consumers typically had two thinking styles on food choice: rational and intuitive. Rational-thinking consumers paid more attention to information search and thoughtful analysis of nutritional information for purchase decision-making than consumers who potentially relied on intuitive thinking. Meanwhile, consumers’ existing label knowledge would increase their visual attention to labels and ultimately improve their purchase decision.
Samant and Seo (2016) [73]	Chicken drumsticks and breasts	Eye-tracking	Consumers with a higher degree of label comprehension looked at the label claims associated with sustainability and process more often and longer than those with a lower degree of label comprehension.
Piqueras-Fiszman et al. (2013) [34]	Jam	Eye-tracking	The ridged surface of the jars could spread consumers’ gaze to another packaging area, such as the border or flavour label. A rounded jar directed consumers’ attention to the flavour label. In addition, the results suggested that marketers should replace textual information regarding the ingredients with visual information on the front of the packaging to influence consumers’ purchase intention.
Bialkova et al. (2014) [74]	Yogurt	Eye-tracking	Consumers had more-prolonged and -frequent fixations in products with traffic-light-colour-coded Guideline Daily Amounts (GDAs) than monochrome GDAs or Choices logos.
Bogomolova et al. (2020) [75]	Orange juice and tomato sauce	Eye-tracking	Better label design could attract consumers’ attention and eye fixation, particularly when the unit price is colour highlighted and for consumers who are less price concerned.
Peschel et al. (2019) [76]	Chocolate	Eye-tracking	Large and high-salience organic labels could engender a higher eye fixation than small and low-salience ones.
Ares et al. (2013) [33]	Mayonnaise, pan bread, and yogurt	Eye-tracking	Regardless of the types of products and label design, selected label zones such as the brand, the ingredients, the image on the label, and the nutritional information always attract consumers’ attention.
Lombard et al. (2020) [77]	Red meat	Eye-tracking	Brand information significantly influences consumers’ purchase intention. An unfamiliar beef brand can only attract higher-educated, younger, and higher-income consumers.
Lombard (2022) [78]	Red meat	Eye-tracking	South African consumers mostly paid attention to the butchery’s name, overlooking the packaging date, sell-by date, and cut name labels. When looking into price labels, younger consumers were more inclined to pay attention to the price labelling aspects, and consumers with a greater level of education paid better attention to price labels since they had better labelling knowledge. Moreover, the influence of brand information is connected with price information.
Brown et al. (2012) [79]	Soft drinks	EEG	Price was crucial for persuading consumers to change from a preferred manufacturer brand to a less-familiar private-label brand when the taste was perceived to be the same.
Helmert et al. (2017) [80]	Onion, bread, sausage, cucumber, tomato, and mushroom	Eye-tracking	Consumers declined suboptimal products compared to impeccable products. However, when presented with differently designed price badges, there is a positive trend to purchase the suboptimal items.
Ballco et al. (2019) [81]	Yogurt	Eye-tracking	Nutrition claims (NCs) on food labelling were an effective way to attract consumers’ attention as consumers care about their health. Within all the NCs, consumers attached the highest importance to fat-free, source of vitamin B6, and source of calcium and the least to low sugar.
Ballco et al. (2020) [82]	Yogurt	Eye-tracking	Nutrition claims and health claims positively affected consumers’ preferences and purchasing decisions, and consumers were more willing to buy products that carry nutrition claims and health claims. However, while consumers like the idea of health claims, they do not want to be overwhelmed by label information.
Oliveira et al. (2016) [83]	Regular and functional probiotic milk	Eye-tracking	Consumers’ attention to labels and purchase intention decreased as information density increased.
Tórtora et al. (2019) [31]	Cookie and cracker	Eye-tracking	While nutritional warnings could attract consumers’ attention, they also discourage consumers’ choice of products.
Van Loo et al. (2015) [84]	Coffee	Eye-tracking	Increased attention to sustainability labels on coffee products was associated with an increased willingness to pay for products that carry those labels.
Meyerding and Merz (2018) [85]	Apple	Eye-tracking	The presence of an organic label positively affected consumers’ trust in food products.
Liu et al. (2022) [86]	Wine	Eye-tracking and AFEA	The country-of-origin (COO) information fetched consumers’ attention. The COO could draw more attention if presented as a logo rather than a script or text.
**Scenario: consume food products**			
Le Goff and Delarue (2017) [87]	Insect-based chips	AFEA	Consumers’ acceptance of consuming insect-based chips is significantly less negative than self-reported.
Gunaratne et al. (2019) [88]	Chocolate	IRTI, GSR, and AFEA	Consumers preferred sweet chocolate more. Salty chocolate could even cause sad emotions.
Horska et al. (2016) [89]	Wine	EEG and AFEA	Facial expressions (happiness, sadness, disgust, neutral emotions, anger, and surprise) can be captured immediately after tasting tested wine samples.
Viejo et al. (2019) [90]	Beer	EEG, AFEA, and IRTI	People do not like bitter beer. A decrease in heart rate occurred when consumers tasted beer samples with higher bitterness.
Mehta et al. (2021) [91]	Energy drink	AFEA	Traditional self-reported emotional measurements and automated AFEA can vary in their outcome; however, all these reactions provide meaningful insights into the differentiation of the products.
Kostyra et al. (2016) [92]	Ham	AFEA	Sensory factors, such as taste and flavour, affected consumers’ emotional responses. Sweet taste could evoke happiness; a bitter taste would cause anger and disgust, and salty and sour tastes would make people feel surprised, sad, and even afraid.

#### 3.2.1. Capture Consumers’ Responses When They Are Watching Promoted Information

Consumers watch promoted information in advertisements, e.g., pictures, videos, and texts, all the time. Biometric approaches have been utilised to investigate how to catch consumers’ attention effectively, which may help with food marketing campaigns.

In 2009, Stockburger et al. (2009) [41] used EEG to check whether vegetarians’ negative perceptions of meat turn corresponding visual stimuli into efficient attention catchers. It was found that meat stimuli are efficient attention catchers for vegetarians. An eye-tracking study conducted by Hummel et al. (2017) [42] aimed to investigate the effect of different food stimuli on visual attention, with participants viewing a series of food pictures varying in calories and convenience. The results showed that cut-up, ready-to-eat, low-calorie food attracted more visual attention than unprepared, low-calorie, and high-calorie food. Men were found to pay more attention to high-calorie food, while women paid more attention to low-calorie food. Viejo et al. (2018) [43] assessed beer samples’ acceptability using biometric technologies (AFEA, eye-tracking, and IRIT). Specifically, the research team predicted the liking level of some sensory attributes of beer (e.g., flavour, foam, bubbles, and carbonation) during beer-pouring videos based on the analysis of participants’ biometric responses (temperature, heart rate, facial expression, fixation number, fixation duration, and pupil dilation). It was found that consumers preferred beers with a medium level of foam height and considered beers with a low foam height as non-desirable, with the lowest liking and lowest perceived quality score. An eye-tracking system was implemented by Motoki et al. (2018) [44] to track and evaluate participants’ eye movements elicited by potato chip food images. It was found that communicating the hedonic components of foods, e.g., a salient stimulus such as by using a delicious-looking picture, can effectively capture automatic visual attention. 

#### 3.2.2. Capture Consumers’ Responses When They Are Wandering in Retail Shops

When consumers wander around a food retail shop, food products will be glanced over while consumers are walking around display shelves. What elements will influence consumers’ visual attention during this process? Researchers mainly investigated this question using an important biometric approach, eye-tracking technology. For example, Reutskaja et al. (2011) [45] found that consumers were good at optimising within a set of items they saw during the search process under time pressure. Thus, through eye-tracking methods, factors influencing consumers’ attention and, consequently, their purchase intention/decision were discussed. These factors included product attributes, such as package design, size, appearance, promotional information, and how the food was displayed on a shelf, such as display location and the number of facings. All the studies included in this section utilised eye-tracking technologies. 

Several studies have investigated the influences of different product attributes, including intrinsic attributes (visible component, appearance, and size) and extrinsic attributes (package design and promotion information), on consumers’ attention and decision-making attitude. For example, focusing on intrinsic attributes, Mitterer-Daltoé et al. (2014) [46] used eye-tracking methods to investigate what underlies consumers’ perceptions of the healthiness of different fish products. By analysing participants’ first eye fixation, first fixation duration, fixation length, fixation count, observation count, and observation length, the study revealed that consumers focused more on a “new or different presentation” to decide whether the dish was more or less healthy, with unusual presentations and fried products being perceived as being less healthy. Further, in 2016, when studying the impact of fat content on consumers’ visual attention and choice of red meat products, Banović et al. (2016) [47] investigated participants’ visual attention to beef. They found that visible fat had a negative relationship with consumer perception. Another article discussing the influence of intrinsic product attributes was conducted by Jaeger et al. (2018) [48]. Eye-tracking was adopted to measure consumers’ attention to and attitudes toward apples with or without defects. It was found that a food product’s appearance was one of the most-important factors influencing consumers’ purchase intention. Consumers were more willing to buy unblemished fruit without any bruises. 

Compared with the limited number of studies using visible intrinsic attributes, research exploring the impact of extrinsic attributes has been more extensive. At the beginning of the century, to explore their in-store buying decision-making processes, Clement (2007) [49] measured consumers’ visual attention and reported that consumers exhibited a muddled search strategy when shopping in grocery shops. It was found that distinctive package designs significantly influenced consumers’ in-store buying decision process. Therefore, the author recommends that marketers constantly have new or distinct packages. More recently, Vriens et al. (2020) [50] investigated how attentional bias and cues impacted consumers’ choices and found that directional cues, such as promotion, significantly affected consumers’ attention and, finally, increased the chance that a consumer would decide to buy. Participants’ decision-making process of purchasing food products at the grocery store was also tracked by Zuschke (2020) [51]. They found that the size and visual salience of products on shelves could attract consumers’ attention and influence their choices. Furthermore, a study by Monteiro et al. (2020) [52] illustrated that consumers’ attention paid to the wine bottle is a determinant of their purchase intention. 

In the meantime, research indicated the crucial role a product’s positioning on a display shelf played in drawing consumers’ attention, with studies by both Zuschke (2020) [51], as previously mentioned, and Bialkova et al. (2020) [53] supporting this conclusion. In 2017, Gidlöf et al. (2017) [54] identified that product packages and displays, including the number of facings, visual salience, and product placement, could positively catch consumers’ attention. Moreover, Drexler and Souček (2017) [55] assessed consumer attention across five product categories (packaged vegetables, dairy food, packaged fish, packaged meat, and frozen food) displayed in store bays and shelves. They found that, except for vegetables, shelf level significantly influenced the variability of attention for all product categories. Specifically, eye-level shelves (4–5 ft) received the most-significant consumer attention, followed by touch-level shelves (3–4 ft). In contrast, stretch-level shelves (about 6 ft) and stop-level shelves (below 3 ft) received the lowest attention. 

#### 3.2.3. Capture Consumers’ Responses When They Are Looking at Food Packaging

After being attracted by various product factors, consumers typically notice some details of its packaging before their next move. Biometric approaches were also used for capturing consumers’ responses in this scenario to understand what kind of package design can attract consumers’ attention and then drive consumers’ purchase decisions. Based on the widely accepted dichotomy of the functions of attention (top-down and bottom-up) [93], articles in this section can be divided into two categories separating studies focusing on either bottom-up factors or top-down ones. Bottom-up stimulus-driven factors refer to characteristics of products that influence consumers’ behaviour, such as packaging colour, decorative font, shape, and image (e.g., image background, location and content, etc.). These factors can contribute to the “visual salience of an object concerning its background or other nearby objects” [50]. Meanwhile, top-down factors are consumers’ existing concepts of or ideas about the products, such as consumers’ emotional responses and cultural backgrounds [33]. 

Outside the categories of top-down and bottom-up factors, López-Mas et al. (2022) [56] tested the usefulness of co-creation with consumers for packaging design on a very general level using a combination of different biometric technologies (eye-tracking, GSR, AFEA). According to the study results, marketers were highly encouraged to engage consumers in all stages of new product design since co-created packages better suited consumers’ needs than those without consumer co-creation. As a result, the product’s success in the market increased.

##### Bottom-Up Stimulus-Driven Factors

The results discussed in Section 3.2.2 outlined the critical role a range of product attributes, including intrinsic (e.g., visible component, appearance, and size) and extrinsic ones (e.g., package design and promotion information), play in attracting consumers’ attention and, consequently, influencing their purchase intention/decision in a retail shop context. The research conducted by Horská et al. (2020) [57] through eye-tracking of consumers’ responses to the packaging of cheese products provided a reasonable explanation for this. Consumers pay attention to factors such as the brand name, the type of product, specific features, the origin of ingredients, and the aesthetic side of design when they look at product packaging.

Specifically, since it is widely accepted that consumers’ attitudes about and preferences for food products can be influenced by visual design elements [60], numerous articles have discussed bottom-up factors from this perspective. The following research was conducted to understand what aspects of food packaging design could attract consumers’ attention to provide a positive first impression, including packaging colour, decorative font, shape, and image (e.g., image background, location and content, etc.). 

Using an eye-tracking approach, Jantathai et al. (2013) [58] studied consumers’ perception of sensory colour and revealed that the colour of food products significantly affects consumers’ gazing behaviour and purchase choice. Through the same technology, Varela et al. (2014) [59] further found that, when there was abundant information on food packages, colour was one of the factors attracting most of the consumers’ attention, together with brands, product names, and graphics. In contrast, consumers paid less notice to health information. Later, in 2017, Husić-Mehmedović et al. [60] applied eye-tracking to explore which package features (e.g., size, packaging colour, shape, and brand name and logo) on the beer cans influenced consumers’ attention and subsequent buying intention. They found that colour and semantic features were the two most-decisive packaging features inducing purchasing. In addition, a recent study has emphasised yellow as a particular colour option to boost consumers’ purchase intention. This type of work has also been performed by Clark et al. (2021) [61], who assessed consumers’ emotional responses to milk packaging samples that exhibited different materials/colours and no label by applying AFEA. It was found that a yellowish colour, decorative fonts, and curved shapes are well associated with a feeling of happiness and purchasing intention. García-Madariaga et al. (2019) [62] described a bottom-up factor connecting colour and images in the packaging. Utilising both EEG and eye-tracking, the research team examined consumers’ attention and declarative preferences regarding three main different attributes as isolated variables: images, texts, and colours, and observed that, between the three attributes, a centrally positioned and colourful product image was the most-important one.

Much literature has paid attention to how to design images for food packaging. By year order, in 2015, a study testing whether the background saliency of food item images displayed on mobile devices affected participants’ attention and impulse buying was conducted by Zhang and Seo (2015) [63]. Eye-tracking technology measured participants’ reactions to donut, taco, and cake pictures. It was found that background salience affected participants’ visual attention. Specifically, the more complex and salient the table setting and decoration, the less attention consumers paid to the food items. Imagine location is also a crucial factor that has been studied. Rebollar et al. (2015) [64] used an eye-tracking device to identify consumers’ patterns of viewing chocolate snack packages. It was found that consumers preferred the logos in the upper left corner of the packaging. In 2018, to investigate how different spatial representations (foreground and background) of a product image on the package could affect consumers’ emotional states and intention to buy the product, Vergura and Luceri (2018) [65] used AFEA to analyse participants’ emotional reactions to various packaging design elements for lemon cake, biscuits, and focaccia snacks. It was found that, compared to the background representation, the foreground representation of products elicited higher favourable emotional reactions because there is less perceived psychological distance between the subject and the product. However, there was no significant difference in the purchase intention between the foreground and background conditions. 

In 2020, Lacoste-Badie et al. (2020) [66] focused on the front side of the food package to investigate the influence of front-of-pack (FOP) variations on consumers’ gazing behaviour. Findings based on data measuring participants’ fixation duration, fixation counts, number of fixations, and eye movements when they viewed biscuit packages through the eye-tracking approach showed that the FOP drew consumers’ attention. Specifically, when food images were displayed vertically, the package attracted a greater fixation time and count than those presented horizontally. A study in 2020 introduced a reasonably specific finding regarding packaging images. Fazio et al. (2020) [67] used eye-tracking to test different packaging combinations to verify the presence of possible weak points in packaging. It was found that olive oil images incorporating a hand using the product could attract consumers’ attention. 

Details in the packaging were captured by researchers as well. For example, Hurley et al. (2015) [68] used eye-tracking to investigate if the different printing methods (digital and flexographic) affected consumers’ decision-making while shopping in the supermarket. Surprisingly, they found no significant differences in consumers’ attention when they shopped for fruit drinks with digital or flexographic labels. 

##### Top-Down Stimulus-Driven Factors

A few researchers have investigated the role of top-down factors on consumers’ purchase intention. These factors include consumers’ emotional responses, cultural backgrounds, shopping goals, and prior knowledge. For example, Samant et al. (2018) [69] used eye-tracking technology to identify the influence of food neophobia on Caucasian consumers’ visual attention toward packaging images and sensory acceptance of ethnic or ethnic-flavoured foods. The findings showed that food neophobia affected consumers’ expected and actual likings of ethnic-flavoured potato chips, especially Chinese-flavoured ones. To better understand the link between consumers’ emotional responses and their eye fixation patterns for a specific area of interest, Gunaratne et al. (2019) [70] used eye-tracking technology and AFEA to explore participants’ gazing patterns and emotional reactions. This study was performed when consumers were exposed to six novel and six familiar chocolate packages. They found that fixations on familiar chocolate packaging were correlated with happiness. To ascertain how culturally distinct consumers from Northern Europe and Northeast Asia perceive and choose product packaging differently, Ploom et al. (2020) [71] adopted an eye-tracking approach to record and analyse participants’ time to first fixation and emotional reactions to biscuits. They found that European and Asian consumers have different perceptions of packaging design and choices and suggested that marketers adapt packaging design accordingly. In addition, a study by Bialkova et al. (2020) [53] revealed that consumers’ shopping goals and prior knowledge also played a role in determining which product got the most attention and was more often chosen. 

#### 3.2.4. Capture Consumers’ Responses When They Are Reading Food Labels

After locating a product, some consumers will read food labels, as an essential information source on the food packaging, to help them make a purchase decision. However, not every consumer does this, as different thinking styles and knowledge levels drive consumers’ behaviour. By employing eye-tracking, Ares et al. (2014) [72] analysed the mean gaze durations and proportions of participants’ gazing at an area of interest. They discovered that consumers typically had rational and intuitive thinking styles on food choices. Rational-thinking consumers paid more attention to the information search and thoughtfully analysed nutritional information when making purchase decisions than consumers who relied on intuitive thinking. It has been reported that consumers’ existing label knowledge increased their visual attention to labels and ultimately improved their purchase decision [73]. Using eye-tracking, consumers with a higher level of label understanding looked at sustainability- and process-related claims more frequently and for longer periods than those with a lower level of label understanding. This result suggested that enhanced label knowledge increases consumers’ visual attention to labels with a possibility of translation into positive purchase behaviour.

Researchers have also investigated how best to efficiently present labels to communicate information from manufacturers to consumers. For example, Piqueras-Fiszman et al. (2013) [34] used combined eye-tracking and word association to collect attentional information and freely elicited associations from participants in response to changing specific attributes of jam. The authors found that the ridged surface of the jars could spread consumers’ gaze to other packaging areas, such as a border or flavour. A rounded jar directed consumers’ attention to the flavour label. In addition, the results suggested that marketers should replace textual information regarding the ingredients with visual information on the front of the packaging to influence consumers’ purchase intention. The label’s design is also essential to attract consumers’ attention. Bialkova et al. (2014) [74] used eye-tracking to explore whether and how attention to nutrition information mediates consumers’ choices. It was revealed that consumers had more prolonged and frequent fixations to products with traffic-light-colour-coded Guideline Daily Amounts (GDAs) than to products with monochrome GDAs or Choices logos. In addition, Bogomolova et al. (2020) [75] tested how unit price and label design factors (position, font size, signposting, and colour highlighting on the price label) could affect consumers’ eye movements during their product decision process. They found that better label design could attract consumers’ attention, particularly when the unit price was colour highlighted and for consumers concerned about the lower price. Peschel et al. (2019) [76] utilised eye-tracking to investigate how the size and salience of an organic label influenced participants gazing patterns and further purchase choices. Through tracking participants’ decision-making process, Peschel and his colleague pointed out that large and high-salience organic labels could engender a higher eye fixation than small and low-salience ones. 

Food labels can reflect significant quantities of information, e.g., nutrition claim and health claim, nutrition content and warning, brand and price, etc. Therefore, a substantial corpus of literature has employed biometric approaches to explore what kind of label information receives the most attention from consumers when they read food labels. Labelling information about brands and nutrition was always important to attract consumers’ attention. This result has been supported by studies conducted by Ares et al. (2013) [33] and Bialkova et al. (2020) [53]. Through eye-tracking, the former research found that, regardless of product and label design types, selected label zones such as brand, ingredients, the image on the label, and nutritional information always attracted consumers’ attention. The latter reported that factors determining consumers’ attention and purchase decision include the brand, nutritional information, and colour-coded labels. 

Biometric studies regarding specific label information have also been conducted over the past ten years. This information included brand, price, health claim (or called a functional label), nutrition claim, nutrition warning, sustainable or organic attributes, and country-of-origin. Lombard et al. (2020) [77] investigated consumers’ attention towards brand labels of beef products using eye-tracking technology to visualise data in the form of a gaze plot and heat map. The results reflected brand information’s significant influence on consumers’ purchase intention by reading brand labels. They revealed that an unfamiliar beef brand attracted more-educated, younger, and higher-income consumers. Recently, the same researcher intended to identify how beef consumers pay attention to the information on price labels with the help of eye-tracking techniques. Surprisingly, they found more evidence for the importance of brand labels [78], as the results showed that South African consumers mostly paid attention to the butchery’s name, overlooking the packing date, sell-by date, and cut name. When looking into price labels, while younger and higher-educated consumers pay greater attention to price labels, the former may be driven by budget constraints or limited financial resources. In contrast, the latter may be due to their better understanding and knowledge of label information. It was also found that the influence of brand information is connected with price information. Using EEG to record consumers’ electrical brain activity, Brown et al. (2012) [79] investigated consumers’ willingness to switch from a preferred manufacturer brand to an unfamiliar private-label brand if the taste was perceived as identical. The findings showed that price was the most-crucial characteristic for persuading consumers to change from a preferred manufacturer brand to a less-familiar private-label brand when the taste was perceived to be the same. Coincidentally, Helmert et al. (2017) [80] used eye-tracking technology to investigate consumers’ eye movement on impeccable and visually suboptimal food items in a purchase or discard decision task. It was found that consumers preferred impeccable products, but when products were presented with differently designed price badges, there was a positive trend to purchase the suboptimal items.

Several researchers have investigated the role of label information on health, function, and nutrition. After knowing what consumers say about their preferences regarding nutrition claims is not reflected in their trade-offs in the marketplace through conducting a choice experiment, Ballco et al. (2019) [81] utilised eye-tracking to help collect participants’ eye movement data to investigate consumers’ preferences for alternative nutrition claims (fat-free, low sugar, high fibre, source of vitamin B6, and source of calcium). It was found that nutritional claims on food labelling effectively attracted consumers’ attention as consumers cared about their health. Within all the NCs, consumers attached the highest importance to fat-free, source of vitamin B6, and source of calcium and the least to low sugar. In 2020, the same research team (Ballco et al.) [82] used the same biometric methods to explore the effect of nutrition and health claims. Consumers were more willing to buy products with nutrition and health claims. However, while consumers like the idea of health claims, they do not want to be overwhelmed by label information. By employing eye-tracking technology, Oliveira et al. (2016) [83] captured participants’ eye movements while they looked at probiotic milk labels and found that their attention to labels and purchase intention decreased as information density increased. In the meantime, unlike consumers’ favourable preference towards nutrition claims, Tórtora et al. (2019) [31] investigated how consumers’ visual processing of nutrition warnings would influence their buying through eye-tracking. They found that, while nutritional warnings could attract consumers’ attention, they also discouraged consumers’ choice of products. 

Food labels can express a product’s unique features, for instance sustainable or organic attributes and country-of-origin. Biometric approaches have been used to evaluate using feature-related symbols and claims and their relation to consumers’ food purchasing decisions. In terms of information about sustainability, Van Loo et al. (2015) [84] explored how consumers visualised sustainable information when they made food choices. Through analysing data collected by eye-tracking, the team found that increased attention to sustainability labels on coffee products was associated with increased willingness to pay for products that carry those labels. Using a similar methodology, Meyerding and Merz (2018) [85] found evidence that an organic label positively affected consumers’ trust in food products. Furthermore, by employing eye-tracking and AFEA, Liu et al. (2022) [86] evaluated the influence of origin information on Pinot Noir wine labels on consumers’ purchase intention. It was found that the country-of-origin (COO) information attracted consumers’ attention and that the COO drew more attention if presented as a logo rather than as in script or textual form.

#### 3.2.5. Capture Consumers’ Responses When They Are Consuming Food Products 

Biometric approaches are commonly used to explore consumers’ acceptance/preferences for certain food products based on consumers’ physiological responses during consumption. Various food products have been studied, including snacks, drinks, and meat.

Le Goff and Delarue (2017) [87] evaluated participants’ facial expressions to study emotional responses to a novel food product: insect-based chips. According to consumers’ self-reported results, consumers have strongly negative expectations of products containing insects. However, the biometric data revealed that consumers’ emotional response while consuming this product was significantly less negative. It is important to note that researchers may integrate different biometric approaches to understand consumers from multiple perspectives. For example, Gunaratne et al. (2019) [88] used eye-tracking and AFEA to assess the autonomic nervous system responses to basic tastes in chocolate to clarify consumers’ preferences. The study revealed that, when participants tasted sweet chocolate, they exhibited a lower level of angry expressions, while they displayed higher levels of sad expressions when tasting salty chocolate. These findings demonstrated that different tastes could stimulate varying emotional responses in individuals. 

In 2016, Horska et al. (2016) [89] used EEG and AFEA to examine participants’ facial expressions and mimic elements when drinking wine. They identified that facial expressions (happiness, sadness, disgust, neutral emotions, anger, and surprise) could be captured immediately after tasting the wine. In this sense, biometrics are valuable tools that will help manufacturers and sellers offer products that truly satisfy customers and not only appear to satisfy them. Viejo et al. (2019) [90] used EEG, AFEA, and IRTI to assess consumers’ acceptability of different beers and created a model to classify beers into liking levels based on subconscious responses. They found that participants did not like bitter-flavoured beer, as there was a decrease in heart rate when they tasted beer samples with higher bitterness. Mehta et al. (2021) [91] explored the emotional reaction elicited by two types of energy drinks to help new product developers and marketing professionals understand consumers’ acceptability of such products. The study concluded that the traditional self-reported emotional measurements and automated AFEA could vary in their outcome; however, all these reactions provide meaningful insights into the differentiation of the products.

To explore consumers’ sensory characteristics of pork products and the effect of the sensory and non-sensory factors on the acceptability of pork products by consumers, Kostyra et al. (2016) [92] used AFEA to measure the facial expressions of participants provoked by the consumption of smoked hams and found that sensory factors, such as taste and flavour, affected consumers’ emotional responses. A sweet taste evoked happiness; a bitter taste caused anger and disgust; salty and sour tastes made people feel surprised, sad, and even fearful. 

## 4. Discussion

### 4.1. Advantages of Utilising Biometric Approaches in Agri-Food Marketing

**Figure 5:**
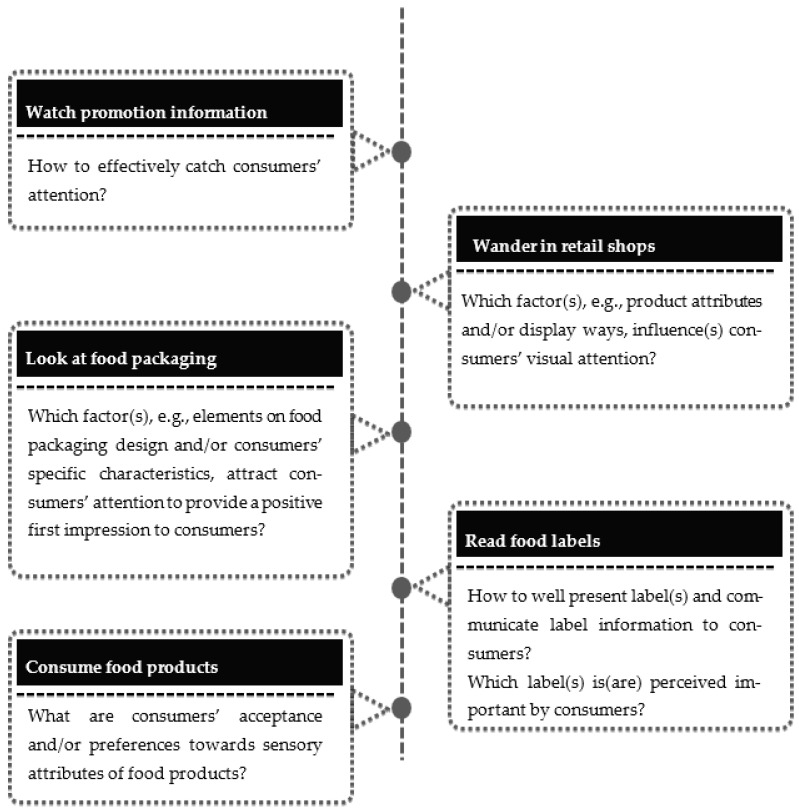
Research questions that can be addressed by biometric approaches across different scenarios of the purchase and consumption behaviour of agri-food products.

Based on the literature, biometric approaches have been widely applied to help researchers understand consumers’ attention and attitude across various scenarios regarding their purchase and consumption behaviour of agri-food products (Figure 5). The rapid development of biometric applications can be attributed to their significant advantages compared with traditional measurements, such as interviews, focus groups, and surveys.

For example, biometric technologies can improve the accuracy and reliability of results by filling the gap of implicit responses left by explicit market research methods [1]. It avoids participants’ consciously controlled answers and gives marketers automatic reactions to products in participants’ subconscious minds [39], which is thought to be connected with the true emotions of consumers. Marketers can better understand consumers’ agri-food purchasing decision-making patterns by reaching the subconscious realm of participants’ minds. In addition, biometric technology can improve the objectivity of the data [94]. Since biometric approaches can directly reveal the real-time reactions of participants, researchers do not need to be mediators to interpret the collected data, where interpretation is highly likely to fall into the pitfall of subjectivity.

### 4.2. Future Trends and Challenges of Utilising Biometric Approaches in Agri-Food Marketing

Given biometric approaches’ comprehensive utilisation and benefits, possible and desirable industry implications can be expected. Biometric methods can help understand consumers’ insights during multiple new product development (NPD) process stages. It is well known that consumers’ perceptions determine the success of a product launching in the marketplace [95]. For example, biometric techniques can help manufacturers deeply understand consumers’ preferences regarding specific product attributes, such as sensory attributes (e.g., appearance and taste), size, package colour, and the way of displaying on the shelf. It is essential to observe the growing trend of employing multiple biometric methods together to gain a comprehensive understanding of consumers in recent years compared to the past. As shown in Table 3, early research predominantly relied on single biometric approaches to understand consumer behaviour in the agri-food sector. However, since 2016, there has been a notable shift with an increasing number of studies embracing a multi-method approach. Researchers are increasingly combining two or even three biometric methods to comprehensively understand consumers, particularly when exploring their responses to food consumption in conjunction with sensory results [88,89,90]. Furthermore, by exploring consumers’ subconscious reactions while watching promotional information, looking at food packages, and reading food labels, biometric techniques can help marketers provide appropriate communication strategies (e.g., promotion tactics, packaging design, and label design). In addition, consumers’ cognitive processes can be inferred by using biometric approaches. Exploring factors influencing consumers’ attention can predict their downstream behaviour. These data may help understand consumers’ decision-making process. Moreover, the current biometric studies mainly focus on physical shopping behaviour. However, considering the rapid development of online shopping behaviour [96], further studies regarding online shopping scenarios are expected. 

It Is foreseeable that a productive way of using biometric techniques is to combine them with explicit marketing research methods. While biometric methods have many benefits, downsides exist [97] (Table 4). Firstly, the biometric technique also has data constraints. For example, eye-tracking allows researchers to know what participants are looking at, yet it provides no means of exploring why they are looking at these aspects. However, understanding the underlying reason for eye movement is essential, especially when the participants’ gaze may not coincide with their cognitive process [98]. Secondly, consumers might face practical challenges in participating in biometric experiments [99]. For example, eye-tracking devices need clear and consistent access to monitor eye movements. Participants must keep reasonably still so that their eye movements, such as fixations, smooth pursuits, and saccades, can be tracked. In addition, not everyone’s eye movements can be tracked; an eye-tracking device’s ability to record participants’ eye movements is influenced by contact lenses, glasses, and pupil colour [100]. Thirdly, analysing data generated by biometric approaches is more complex than traditional market research methods and heavily relies on the expertise of researchers [101,102]. While inaccurate data can result from the unskilled implementation of biometric technologies, advanced techniques such as machine learning can help improve the analysis of biometric data and provide more-accurate and -valuable insights [103]. However, the monetary and time cost of applying biometric approaches should be considered, which is the last shortcoming noted by the authors. Significant investment is required to train technicians and purchase and maintain biometric equipment [94]. For instance, many biometric approaches, such as AFEA and IRTI, must be conducted in a highly controlled environment and typically incur a considerable construction or rental fee. Moreover, as an emerging technology, researchers in the biometric fields should always pay close attention to privacy and ethical considerations [104].

## 5. Limitations

While this study has provided valuable insights by reviewing the literature on the topic, it also acknowledges certain limitations. Notably, the inclusion of only English articles might have excluded valuable research available in other languages. To address this limitation in future studies, researchers can consider using translation tools or collaborating with authors proficient in different languages to explore a more-diverse range of databases and enhance the overall understanding of the subject. In addition, although many research and review articles were identified, other article types, e.g., books, book chapters, notes, conference papers, and industry or governmental reports, are all recommended for future studies to explore. 

## 6. Conclusions

This research provided an overall picture of the application of behavioural biometrics in agri-food marketing by synthesising and organising the main findings of existing studies within such areas. 

Despite the interruption of COVID-19, an apparent upward trend can be seen in published articles in the last decade concerning biometric applications in the agri-food area. Geographically, Europe and the USA are the two most-active areas conducting research in this field. Although various biometric approaches have been utilised in the collected studies, nearly half of the research has used eye-tracking. Biometrics is a valuable tool for agri-food marketers to understand consumers’ preferences and behaviour in various scenarios, from purchasing to consumption. It helps avoid cognitive biases, but also faces challenges such as data constraints and high costs. Nevertheless, it can offer far-reaching benefits for the industry, e.g., aiding in new product development, communication strategies, and understanding consumer decision-making processes based on cognitive responses.

The findings demonstrated that biometric techniques are becoming more widely used in the agri-food sector to help practitioners understand consumers through subconscious behaviour responses. This is an encouraging trend, given that biometric approaches can help the industry better understand its consumers when used with traditional explicit measurements. Furthermore, the authors encourage the agri-food research community to investigate applying the existing techniques more comprehensively and explore new technologies as they are developed.

## Figures and Tables

**Table 1:** Exclusion criteria used for eligibility screening.

**Inclusion Criteria**
Research article Review article Full-text papers published in peer-reviewed journals in the English language Articles focusing on the application of biometric approaches in the agri-food sector
**Exclusion criteria**
Opinion papers and outlooks; conference papers and abstracts; concept articles Not related to the marketing area Not related to the agri-food sector Articles focusing on developing technologies, rather than applying them

**Table 2:** The rationale of using common biometric approaches in marketing.

Biometric Approach	Main Features
Eye-tracking	**Raw data**: eye movements (incl. fixation and saccades) and pupil dilation **Analysed data**: gaze patterns, fixation duration, areas of interest **Application rationale**: attention can indicate consumers’ interest and purchase intent
Automatic facial expression analysis (AFEA)	**Raw data**: facial movements (e.g., lips, eyes, cheeks, and mouth) **Analysed data**: estimated emotional valence (e.g., happiness, sadness, surprise, fear, anger, and disgust) **Application rationale**: coherence between consumers’ emotions and facial expressions
Electroencephalograms (EEGs)	**Raw data**: brain cortical electrical activity **Analysed data**: brainwave patterns **Application rational**: associations of consumers’ cognitive responses with brainwave patterns
Galvanic skin response (GSR)	**Raw data**: skin conductance level, short-duration skin conductance responses (SCRs), heart rate (HR) **Analysed data**: GSR peaks, amplitude, SCR onset, and response frequency **Application rationale**: SCL, SCRs, and HR can reflect consumers’ emotional states
Infrared thermal imagery (IRTI)	**Raw data**: body temperature changes **Analysed data**: thermal images, changes in body temperature over time **Application rationale**: different stimuli can elicit consumers’ different natural reactions, including changes in body temperature

**Table 4:** Current downsides of biometric approaches and their potential solutions.

Downsides	Potential Solutions
**Data type**: Limited insight into participants’ underlying cognitive processes	Combine biometric approaches with other methods to deeply understand consumers
**Participation**: Practical challenges for participants in biometric experiments	Innovate equipment and/or recruit qualified participants
**Data analysis**: Complexity in data analysis and reliance on researchers’ expertise	Use advanced techniques (e.g., machine learning)
**Cost**: High monetary and time cost related to equipment, staff, and location	Boost investment from government, institutions, and industry
**Ethics**: Privacy and ethical considerations	Prioritise privacy, obtain informed consent, and ensure data security

## Data Availability

Data is contained within the article.
